# Hemiballismus, Hyperphagia, and Behavioral Changes following Subthalamic Infarct

**DOI:** 10.1155/2012/768580

**Published:** 2012-10-18

**Authors:** Masoud Etemadifar, Seyed-Hossein Abtahi, Seyed-Mojtaba Abtahi, Motahreh Mirdamadi, Sepideh Sajjadi, Aryan Golabbakhsh, Mohammad-Reza Savoj, Mahboobeh Fereidan-Esfahani, Zahra Nasr, Nasim Tabrizi

**Affiliations:** ^1^Department of Neurology, Medical School, Isfahan University of Medical Sciences, Isfahan 81744-176, Iran; ^2^Medical School, Isfahan University of Medical Sciences, Isfahan 81744-176, Iran; ^3^Medical Students' Research Center, Isfahan University of Medical Sciences, Isfahan 81744-176, Iran; ^4^Department of Otolaryngology, Medical School, Isfahan University of Medical Sciences, Isfahan 81744-176, Iran; ^5^Department of Psychiatry, Medical School, Isfahan University of Medical Sciences, Isfahan 81744-176, Iran

## Abstract

The function of subthalamic nucleus (STN) which is a part of the basal ganglia system is not clear, but it is hypothesized that this component might be involved in action selection. Unilateral damage to STN, which can commonly occur due to the small vessel stroke mainly, causes hemiballismus and sometimes hemichorea-hemiballismus. This paper deals with a 60-year-old patient with sudden onset of abnormal movements in his right limbs. He had increased appetite and hyperphagia and also developed mood and behavioral changes (aggressiveness, irritability, anxiety, and sometimes obscene speech). The magnetic resonance imaging revealed infarct area in left subthalamus. In our case, hemiballismus is caused by infarction in left subthalamic area. Occurrence of irritability, anxiety, and some behavioral changes such as aggressiveness and obscene speech can be explained by impairment of STN role in nonmotor behavior and cognitive function as a result of infarct.

## 1. Introduction

The functions of subthalamic nucleus (STN) which is a part of the basal ganglia system is not clear, but it is hypothesized that STN might be involved in action selection [1, 2]. Unilateral damage to STN, which can commonly occur due to the small vessel stroke in patients with diabetes, hypertension, or smokers, mainly causes hemiballismus and sometimes hemichorea-hemiballismus [3–5].

As poststroke movement disorders, chorea and ballismus often coexist and usually involve contralateral limbs. Hemiballismus is usually the manifestation of contralateral STN involvement; however, lesions in the striatum, thalamus, cerebral cortex, subcortical area, and midbrain can also cause hemiballismus [3–5].

STN is involved in motor and cognitive performances through its key role in the basal ganglia-thalamocortical circuits, but mechanisms of these different modality regulations have not yet been elucidated. A number of reports suggested that subthalamic lesion or stimulation can result in cognitive, mood, behavioral, and personality changes, but similar disorders following subthalamic infarction were scarcely observed in the literature [6–11].

Pathological eating behaviour that ranges from decreased to increased appetite, impaired regulation of hunger and satiation signals, hyperphagia, and anorexia are reported in lesions (mostly tumors) involving the ventromedial hypothalamus and STN. Also there is a report of a patient with medial thalamic ischemic stroke that caused compulsive hyperphagia. Moreover, eating disorders may also occur in temporal lobe tumors, temporal lobe epilepsy, and advanced stages of degenerative disease with neuronal loss in the medial temporal lobe and Kluver-Bucy syndrome [6–11].

In this study, we report a patient with left STN infarct presented with right side hemichorea-hemiballismus, mood and behavioral changes and also increased appetite and hyperphagia.

## 2. Case Report

A 60-year-old right-handed man was admitted to our emergency department for sudden onset abnormal movements in his right limbs during the daily activity. The symptoms were aggravated while walking, disturbing his coordination, and also, were alleviated in relaxation and eliminated completely during sleep. He had no involuntary movement on his face or left side and no complaint of weakness or slurred speech. He had increased appetite and hyperphagia and also developed mood and behavioral changes (aggressiveness, irritability, anxiety, and sometimes obscene speech) since the onset of the recent event. He had a past history of hypertension and ischemic stroke (a year before, he had experienced sudden onset of right side hemiparesis which was completely improved after a few months) and was treated with aspirin 80 mg/day. His family history was negative for any significant disease.

General examination revealed no significant abnormal finding. In the neurological examination, mental status was normal. Cranial nerves function was also intact. In examination of motor system, no abnormality was detected in muscle tone; and, force of extremities was normal. Deep tendon reflexes were symmetric, and no pathologic reflexes were found. Involuntary movements, including continuous, arrhythmic and purposeless choreiform movements with occasional brisk proximal ballistic movements were seen affecting both upper and lower limbs in the right side with the same severity. No abnormality was noted in cerebellar or sensory system examination, and also he had no abnormal cortical sensation.

Lab tests including CBC, ESR, biochemistry, and lipid profile were within normal ranges, and cardiac echocardiography showed no significant abnormality or source of emboli.

**Figure 1:**
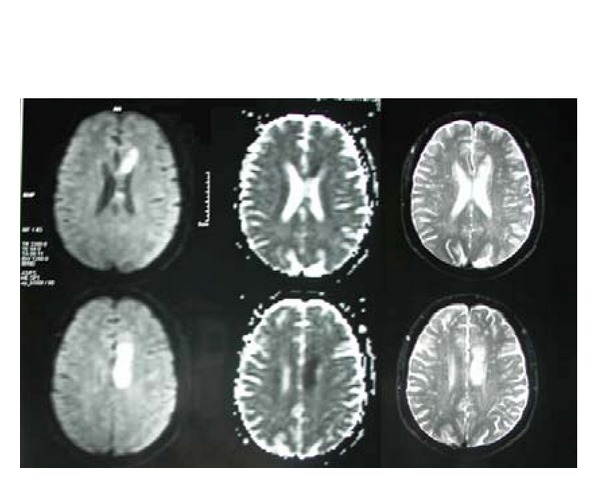
The first MRI shows an infarct in left centrum semiovale near the left lateral ventricle with mismatch in DWI and ADC map imaging and also multiple lacunar infarcts.

**Figure 2:**
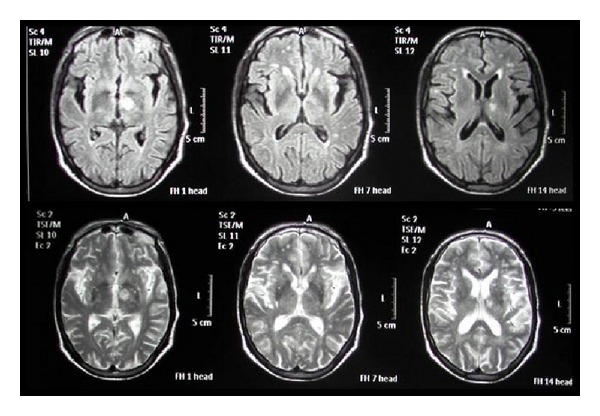
The second MRI (2 days after recent event) revealed another infarct area in left subthalamus.

The brain MRI that was performed in July 2008 (after the first stroke) demonstrated an infarct in left centrum semiovale near the left lateral ventricle with mismatch in DWI and ADC map imaging and also multiple lacunar infarcts (Figure 1). The second MRI in February 2010 (2 days after the recent event) revealed another infarct area in the left subthalamus (Figure 2).

The patient was treated with aspirin 80 mg/day, dipyridamole 75 mg 2 times a day, and atorvastatin 20 mg/day. Also, Keppra (levetiracetam) was prescribed 500 mg 2 times a day to control abnormal movements. The hemiballismus-hemichoreiform movements were improved after a few days and completely resolved after 3 months. Hyperphagia was improved after 10 days, and behavioral changes were subsided a month later.

## 3. Discussion

STN as a regulator of motor function innervates other structures, including very important connections to the inside of the globus pallidus that provides the excitement needed to drive it. 

Overall, the STN connections include both motor and nonmotor constituents. STN originates efferent projections towards both segments of the globus pallidus, substantia nigra, striatum, and pedunculopontine nucleus. Besides, afferent pathways result principally from the lateral pallidum along with three functional units, motor, associative, and limbic, from the cerebral cortex (chiefly, premotor, primary motor, and supplementary motor), the parafascicular thalamic nucleus, the substantia nigra pars compacta, the dorsal raphe nucleus, and the pedunculopontine nucleus. In the literature, regarding the basal ganglia circuitry, a specific function is ascribed to the cortico-subthalamic projection which is also, mentioned as the “hyperdirect pathway.” STN works as the main component of the basal ganglia circuitry. 

On one hand, lesions of STN may lead to movement disorders (hemichorea/hemiballismus). On the other hand, it is generally postulated that in Parkinson's disease and Dopamine agonist depletion states the STN is hyperactive and STN deep brain stimulation (DBS) can improve rigidity and bradikinesia. The two aforementioned parallel points may bring the interesting concept to mind that the STN lesioning may just result in “net movement”; wherein, movement deficits are improved, and “normal” movements are exaggerated [11].

Hemiballismus occurs in only about 0.45 cases per hundred thousand stroke victims. Stroke is the most common cause of hemiballismus. Hemiballismus can also occur as a result of a traumatic brain injury, amyotrophic lateral sclerosis, nonketotic hyperglycemia, neoplasms, vascular malformations, tuberculomas, demyelinating plaques and complications from HIV infection such as toxoplasmosis [3]. In our case, hemiballismus is caused by infarction of the left subthalamic area. It was traditionally thought that hemiballismus was only caused by injury to the subthalamic nucleus, but new studies are showing that damage to other areas of the brain can also be responsible for causing this disorder. However, hemiballismus caused by lesions in the subthalamic nucleus is more severe than other causes [1–5].

STN has a potent regulatory function in processing of associative and limbic information towards cortical and subcortical regions. Involvement of STN in physiological and pathological nonmotor behavior has now largely been established. Clinical observations in patients suffering from Parkinson's disease treated with DBS of the STN show that these patients may suffer from postoperative changes in nonmotor behavior mainly involving alterations in cognitive functions [6–10].

Several studies have been done to investigate the effects of STN DBS on mood, memory, and behavior. Some showed variable effects on working memory (WM) and response inhibition (RI) performance and some reported cases with aggressive impulsive episodes, suicide and suicidal attempts, apathy, severe depression, psychosis, and hypomania following bilateral STN DBS. Another study revealed psychosis, worsening of depressive symptoms, increasing in anxiety, worsening in depression following implantation, and also a relevant improvement in mood and anxiety symptoms after the surgery. Also cases of mania and depression have been reported after STN DBS [7–10]. Of note, the jury is still out as to whether DBS behaves as a lesion does; in other words, the outcome of DBS method may not exactly fit to that of a lesion, and this point may limit the validity of some postulations in this regard.

In our case, STN infarct was demonstrated by irritability, anxiety, and some behavioral changes such as aggressiveness and obscene speech, which can be explained by impairment of STN role in nonmotor behavior and cognitive function.

Conventionally, the basal ganglia and cortico-limbic regions are the areas for anatomical substrates of eating disorders. In diencephalic region, hyperphagia may result from hypothalamic, thalamo-cortical, or limbic lesions. Lesions involving the ventromedial hypothalamus, the amygdale, and the fiber bundle from the substantia nigra to the basal ganglia alter the signal of satiation and food intake. Eating disorders caused by hypothalamic dysfunction are characterized by dysregulation of hunger and satiation signals and are associated with other endocrine dysregulations. Cortical lesions also may cause eating disorders if they involve the temporal and frontal association areas connected to the basal and diencephalic systems. Moreover, clinical observations and animal studies suggest that the limbic structures and their connections are strongly involved in the regulation of appetite [8–10]. Seemingly, STN has a part in the processing of motivation, and such an assertion is mainly based on the anatomical notion that STN is a unit of the limbic loop involving the prefrontal cortex, the nucleus accumbens, and the ventral pallidum. Experimental motivation studies have established that STN lesions do not increase hunger, though, they stimulate food motivation. This might explain the mechanisms behind clinical reports on “hyperphagia” induced by STN lesions [11].

In our patient, we believe that transient thalamo-cortical dysfunction due to impairment of the connection between the medial thalamic nuclei and the frontal or temporal lobes may have been the determinant of the compulsive hyperphagia.
